# Smart and Multifunctional Nanomaterials and Applications for Food Safety

**DOI:** 10.3390/bios13100928

**Published:** 2023-10-16

**Authors:** Long Wu

**Affiliations:** 1 Hainan Engineering Research Center of Aquatic Resources Efficient Utilization in South China Sea, Key Laboratory of Seafood Processing of Haikou, School of Food Science and Technology, Hainan University, Haikou 570228, China; longquan.good@163.com; Tel.: +86-0898-66193581; 2 Key Laboratory of Tropical Fruits and Vegetables Quality and Safety for State Market Regulation, Hainan Institute for Food Control, Haikou 570314, China

Due to growing concerns about food safety and public health, the contaminants or residues of various harmful substances in food have received much attention in recent years [1,2,3]. With the increasing complexity of the food supply chain and the expansion of the scale of food production, more stringent regulations and standards are now required for food quality and safety [4,5,6]. Thus, it is indispensable to develop an efficient detection method to analyze all kinds of foodstuffs. Currently, the most commonly used detection methods, including high-performance liquid chromatography (HPLC), gas chromatography (GC), gas chromatography–mass spectrometry (GC-MS), and liquid chromatography–mass spectrometry (LC-MS), are demonstrated to have exceptional sensitivity and selectivity, but suffer from limitations such as being time-consuming procedures requiring expensive equipment and professional operations [7,8,9,10]. Fortunately, the advancement of smart and functional nanomaterials endows rapid detection methods with flexible construction strategies and facile applications, which include Raman technique-based [11,12], electrochemical [13], and electrochemiluminescence analyses [14], fluorescence measurement [15,16] and immunoassays, and so on [17,18]. By adopting nanomaterials, the sensitivity and accuracy of rapid detection methods can be greatly enhanced, making them highly applicable in the field of food safety analysis.

In this Special Issue, we focused on advances in nanomaterial-modified rapid detection methods for food safety detection and analysis. With the rapid development of nanotechnology, all kinds of new nanomaterials, including organic nanomaterials, fluorescent nanomaterials, nanozymes, and carbon-based nanomaterials, have been adopted in analytical methods [19,20]. Newly emerging nanomaterials have high specific surface areas, enabling higher catalytic and sensing responses as well as better optical, magnetic, and electrical performance [21]. Also, such nanomaterials can serve as smart and functional elements in many tools, such as in microfluidic assay devices (µFADs) [22], micro-electromechanical systems [23], optical sensors [24,25], force or pressure sensors [26], magnetic relaxing sensors [27], etc. The application of such nanomaterials can greatly enhance the performance of biosensors, including their sensitivity, selectivity, and accuracy, thereby advancing the development of food safety detection technologies.

This Special Issue has collected a diversity of studies that focus on smart and functional nanomaterial-fabricated biosensors in food safety. It includes four review articles, three communications, and six research articles, which I briefly describe in the next paragraphs. This Editorial aspires to describe the advances of nanomaterials in food safety applications by covering all kinds of analytical methods for detecting food contaminants.

Ochratoxin A (OTA) is stabilized well in food and poses potential threats to human health, including nephrotoxicity, hepatotoxicity, teratogenicity, and carcinogenicity. In this Special Issue, Nawaz et al. conducted a comprehensive study on zinc selenide (ZnSe) nanostructures to construct an aptasensor for the detection of OTA in food. They synthesized six types of ZnSe nanostructures, including nanorods, μ-spheres, and nanoclusters, and further evaluated the fluorescence bursting efficiency of ZnSe nanostructures. Finally, multifunctional ZnSe-B6 nanostructures with high negative charges were synthesized and employed to develop a fluorescent aptasensor for the detection of OTA with a wide linear range of 0.1 to 200 ng/L and a limit of detection of 0.07 ng/L. In addition, Yang et al. constructed a guanine-quenched fluorescence sensing platform for the detection of OTA, which relies on monitoring fluorescence changes resulting from conformational alterations of the nucleic acid aptamer upon binding to OTA. Another research proposed by Yang et al. focused on exploring the fluorescence quenching ability of G-quadruplexes based on photo-induced electron transfer, wherein G-quadruplexes acted as quenchers and as a sensing platform for OTA and potassium ions (K^+^). During the recognition process, the formation of the G-quadruplex structure occurs, causing the quenching of the labeled fluorescein fluorophore (FAM), thereby enabling the detection of OTA with a limit of detection (LOD) of 0.19 nM. All of the above research works are based on the principle of fluorescence quenching and show distinct fluorescent strategies, offering flexibility in the sensitive detection of OTA in food safety and environmental monitoring.

The rapid development of nanomaterials, metal–organic frameworks (MOFs), carbon dots (CDs), and metal nanoparticles in recent years has brought about new directions for fluorescence analysis. CDs have found broad applications in biomedical imaging and biosensors due to their unique optical properties, biocompatibility, affordability, sensitivity, and ease of functionalization. Xu’s article constructed fluorescent probes based on red carbon dots for the detection of Mn^2+^ and Zn^2+^ in macroalgae. The results showed good linearity between fluorescence intensity and concentrations of Mn^2+^ and Zn^2+^. In addition, Li et al. designed a lanthanide-based ratiometric fluorescent probe for the determination of bacterial endospore biomarkers. In the study, CDs were bound with europium ions (Eu^3+^) to create Eu^3+^/CDs fluorescent probes. The fluorescence intensity (PL) ratio of Eu^3+^/CDs showed a good linear relationship (R^2^=0.9961) and a low LOD (18.3 μM) for the detection of dipicolinic acid. The ratiometric fluorescent sensor showed great potential for application in complex food matrices.

Silver nanoparticles (AgNPs) and gold nanoparticles (AuNPs) are widely employed to prepare surface-enhanced Raman scattering (SERS) substrates. The SERS signals can be enhanced on the surface of noble metal nanomaterials due to the abundant SERS “hotspots”, thus achieving highly sensitive detection of targets. As an example, Yuan et al. prepared copy paper loaded with AgNPs (AgNP-CP) for the rapid and in situ detection of sodium metabisulfite on shrimp surfaces. Compared to textiles and other flexible materials, copy paper is the most commonly used substrate for loading AgNPs and AuNPs and is favorable for in situ SERS detection. In addition, the AgNP-CP substrates demonstrated improved performance for the sensitive and accurate detection of sodium metabisulfite, which can be further applied to the on-site and non-destructive testing of other contaminants in seafood in the future.

Due to the intrinsic enzyme-like properties of nanozymes, they are widely used in immunosensors to replace natural enzymes like horseradish peroxidase, which holds great potential for the real-time detection of pathogenic bacteria and evaluation of food risk factors. For instance, Lang et al. conducted a review of recent advancements in nanozyme-based methods for the determination of risk factors in food. The authors provide a detailed description of common methods employed for detecting risk factors in food, such as pathogenic microorganisms, toxins, heavy metals, and pesticide residues, and explain the principles and applications of nanozymes in immunosensors. The review provided new insights into developing different nanozyme-based sensors for food safety analysis, especially innovating the structure of immunosensors.

Electrochemical sensors are widespread in food safety detection because of their advantages of being low cost, simple to operate, and portable. In this Special Issue, Zhou et al. presented a review of recent advancements and trends in electrochemical sensors based on molecular imprinting technology (MIT). The authors conducted a comprehensive review of molecularly imprinted electrochemical sensors (MIECs) in terms of design, operating principles, and functionality. In addition, the application of MIECs in food and pharmaceuticals safety was discussed, as well as the challenges and prospects for the development of new electrochemical methods. Furthermore, Wu et al. discussed recent advances in bioelectronic sensing based on chitosan-based hydrogels. With different types of chitosan-based hydrogels, including electrode-based hydrogels, conductive materials conjugated hydrogels, ionically conductive hydrogels, and redox-based hydrogels, the authors describe the properties of these materials and their wide range of applications in fields such as medicine and food safety. Wang et al. provided an overview of recent advancements on electrochemical biosensors for food safety detection, highlighting their advantages of miniaturization, low cost, rapid detection as well as high sensitivity and selectivity with the inclusion of nanomaterials. The three review articles on electrochemical sensing offered an in-depth exploration of the prospective contributions of electrochemical biosensors for detecting biological contaminants, chemical pollutants, and genetically modified crops. The reviews have highlighted the significant role of electrochemical sensors in food safety detection, and new breakthroughs have been made to address food safety issues with the aid of functional nanomaterials.

This Special Issue compiles a collection of research articles and reviews on the application of nanomaterials in food safety detection, including electrochemical detection, nanozymes-based immunosensors, fluorescent analysis, and SERS sensors. Using smart or functional nanomaterials, some limitations of those methods are overcome; for example, the conductivity and surface area of the electrode are increased, the loading capacities of antibodies and markers are improved, and the fluorescence intensity and SERS signal become stronger. Still, much more needs to be achieved to meet the needs of different application scenarios with more detection functions, such as on-site, nondestructive, multicomponent, and real-time monitoring. This Special Issue is the collective effort of several authors who have made important contributions to different detection methods on food safety and are paving an avenue towards the development of new analytical methods. 
